# Intra-Rater, Inter-Rater, and Test–Retest Reliability of a Laser- and Inclinometer-Based Hip Joint Position Sense Test in Healthy Adults: A Two-Phase Study with Preliminary Reference Values

**DOI:** 10.3390/muscles5020045

**Published:** 2026-06-19

**Authors:** Joévin Burnel, Benoit Vallee, Benoit Pairot de Fontenay, Joachim Van Cant

**Affiliations:** 1 Research Unit in Rehabilitation Sciences, Faculty of Human Movement Sciences, Université Libre de Bruxelles, Route de Lennik 808, 1070 Bruxelles, Belgium; joachim.van.cant@ulb.be; 2 Conseil Scientifique de TOHA, Thread of Life Prime, 37000 Tours, France; 3 Institut de Formation en Masso-Kinésithérapie de L’Est-Francilien (IFMKEF), 77124 Chauconin-Neufmontiers, France; 4 Ramsay Santé, Clinique de la Sauvegarde, 69009 Lyon, France; 5 Lyon Ortho Clinic Global, 69009 Lyon, France; 6 Interuniversity Laboratory of Human Movement Biology, Claude Bernard University Lyon 1, 69010 Villeurbanne, France; 7 The Running Clinic, Lac Beauport, QC G3B 0W8, Canada; 8 French Society of Sports Physical Therapist (SFMKS Lab), 95270 Asnières sur Oise, France

**Keywords:** hip proprioception, joint position sense, neuromuscular control, muscle function, reliability, clinimetrics

## Abstract

Hip joint position sense (JPS), a key component of neuromuscular function arising from muscle spindle and periarticular mechanoreceptor input, remains underexplored, with no standardized and reliable clinical protocol available to assess hip proprioception. This study evaluated the intra- and inter-rater reliability of a laser- and inclinometer-based active hip JPS protocol and established preliminary references in healthy adults. A two-phase reliability study was conducted in accordance with GRRAS and COSMIN guidelines: 17 participants for reliability analyses and 57 for preliminary references. Six movement directions were assessed (flexion, extension, abduction, adduction, medial and lateral rotations). Reliability was quantified using intraclass correlation coefficients with their 95% confidence intervals, using two-way random-effects models with absolute agreement (ICC(3,1) for intra-rater and ICC(2,1) for inter-rater analyses), interpreted as poor (<0.50), moderate (0.50–0.70), or good (≥0.70). Absolute measurement error was reported as standard error of measurement (SEM%) and 95% minimal detectable change (MDC95%), normalized to target amplitudes to allow direct cross-direction comparison. Intra-rater reliability ranged from poor to moderate, with experienced raters reaching ICC = 0.64 (95% CI [0.39; 0.80]) for medial rotation. Inter-rater reliability improved across sessions, peaking for medial rotation (ICC = 0.78; 95% CI [0.50; 0.91]). Rotational movements yielded the lowest SEM% (3–6%), indicating high measurement precision despite trial-to-trial variability (MDC% 9–31%). Normative errors were largest in flexion (21.4 cm) and smallest in rotations (≈2.2–2.3°). Despite overall low-to-moderate reliability, the protocol achieved clinically acceptable measurement precision (SEM% < 10%) for rotational tasks, whereas the laser-based sagittal and frontal-plane components remained exploratory. The protocol provides preliminary reference values for hip JPS in healthy adults and requires further validation before clinical use.

## 1. Introduction

Proprioception refers to the sense of body position and movement, arising from afferent signals within muscles, tendons, joints, and skin [1,2,3]. It plays a crucial role in motor control, joint stability, postural regulation, and movement planning, allowing precise regulation of voluntary actions and the effectiveness of stabilisation strategies [1,2,3]. Altered proprioceptive function at the hip has been reported in several clinical conditions, including femoroacetabular impingement, early osteoarthritis, and post-traumatic or sport-related injuries [4,5].

In contrast to other joints, particularly the knee, ankle, and cervical spine, the proprioceptive function of the hip has been comparatively underinvestigated [6,7,8,9,10,11,12]. Existing studies have mainly examined vibration perception, while joint position sense (JPS) remains largely unexplored [13,14,15], leaving no standardized and reliable assessment tools for this joint. In cervical proprioception research, active laser-based repositioning tests are widely used, a simple and low-cost method whose reliability has been demonstrated [6,7,8,16]. In parallel, recent work has demonstrated the validity and reliability of digital inclinometers for quantifying hip rotations, showing agreement levels comparable or superior to bubble, smartphone, or optical systems [17,18,19]. Combining these accessible tools enables accurate angular measurement and precise evaluation of repositioning error without specialized equipment. Despite these advantages, such approaches have rarely been applied to the hip and have not yet been validated for this joint. Nevertheless, several studies indicate that targeted proprioceptive training of the hip can enhance motor control and improve specific proprioceptive abilities, including performance on static and dynamic balance tests [20,21].

In this context, tests based on active joint position reproduction using a laser pointer represent a promising approach, as they allow for a standardized, low-cost, and easily transferable method of assessment in clinical settings [16]. However, the intra- and inter-rater reliability of such a protocol applied to the hip has not yet been established, particularly across different movement directions (flexion, extension, abduction, adduction, and medial and lateral rotations). According to the COSMIN initiative (COnsensus-based Standards for the selection of health Measurement INstruments) and the GRRAS guidelines, the appraisal of measurement properties requires a clear distinction between reliability, measurement error, and validity [22,23]. Reliability is defined as the proportion of the total variance in the measurements that is due to true differences between subjects, and is typically quantified by the intraclass correlation coefficient (ICC). Measurement error refers to the systematic and random error of a patient’s score that is not attributed to true changes in the construct to be measured, and is evaluated through the standard error of measurement (SEM) and the minimal detectable change (MDC) [24]. Validity, by contrast, denotes the degree to which an instrument measures the construct it purports to assess [22]. These complementary but methodologically distinct properties require a rigorous clinimetric appraisal, combining relative (ICC) and absolute (SEM, MDC) indices, before any clinical use of a new assessment tool [22].

The primary aim of this two-phase study was to evaluate the intra-rater, inter-rater, and test–retest reliability of a standardized laser- and inclinometer-based protocol for assessing hip joint position error. A secondary aim was to establish normative JPS values in a healthy adult population, providing a broader representation of typical proprioceptive performance and supporting the future development of clinically meaningful benchmarks. To enhance the robustness of inter-rater reliability estimates, assessments in the preliminary reference values were performed by two novice raters on a larger sample.

## 2. Methods

This reliability study was conducted in line with the ethical principles of the Declaration of Helsinki (Fortaleza revision, 2013), the GRRAS recommendations (Guidelines for Reporting Reliability and Agreement Studies), and the COSMIN standards for reliability studies. The protocol was reviewed and approved by the Hospital–Faculty Ethics Committee of Erasme Hospital (Université Libre de Bruxelles, Brussels, Belgium) (Ref. P2024/505/CCB 84062024000361). All participants received detailed oral and written information about the study objectives, procedures, potential risks, and their rights as participants. Written informed consent was obtained from all participants before inclusion.

### 2.1. Participants

Participants were recruited consecutively from the Faculty of Human Movement Sciences at the Université Libre de Bruxelles (ULB) through information posters and word-of-mouth. Inclusion criteria were: age 18 years or older; absence of musculoskeletal disorders or chronic conditions likely to affect the hip; sufficient understanding to follow experimental instructions; and a history of regular sport participation between the ages of 6 and 18 years (at least one session per week for a minimum duration of three years). Exclusion criteria included any history of hip surgery, acute or subacute hip injury within the previous six months, current pregnancy, and the use of medication that could affect proprioception (particularly drugs with neurological, cardiovascular, or endocrine effects).

### 2.2. Study Design

**Figure 1:**
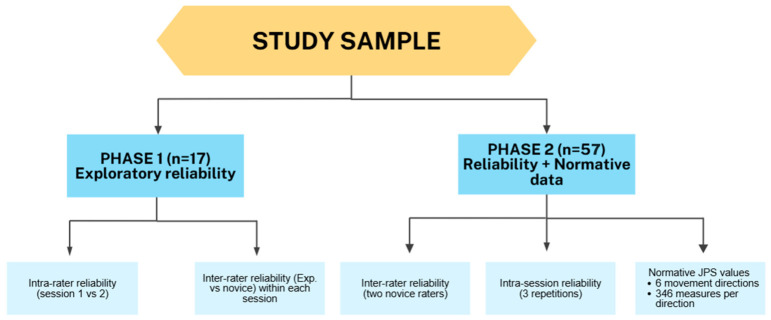
Overview of the study design, participant flow, and reliability analyses performed in Phases 1 and 2. JPS = Joint Position Sense; Exp. = Experienced rater; vs = versus; *n* = sample size.

Phase 1 aimed to evaluate the initial reliability of the JPS assessment protocol in a sample of 17 participants (Figure 1). Two raters (one experienced and one novice) conducted two testing sessions separated by 24 h. This phase allowed the examination of: (1) intra-rater reliability, based on repeated measurements across the two sessions; (2) inter-rater reliability, by comparing the experienced and novice raters within each session.

Phase 2 included 57 participants and aimed to confirm inter-rater reliability in a larger sample by analysing agreement between two novice raters (Figure 1). This phase also incorporated an additional intrasession reliability analysis based on the three repetitions performed in each session. Finally, the data collected from all 57 participants were used to establish normative joint position error values for the six hip movement directions, providing reference thresholds to support clinical interpretation.

### 2.3. Testing Sessions and Rater Allocation

All assessments were conducted in a controlled and standardized environment, across two examination rooms, following identical procedures. In Phase 1, each participant was assessed twice, once during Session 1 (Day 1) and again during Session 2 (Day 2), 24 h apart. The 24 h interval was selected to minimize potential fatigue effects induced by testing while ensuring stability of the proprioceptive construct between assessments [25]. This design enabled the evaluation of intra-rater reliability by comparing repeated measurements across the two sessions for both the experienced and novice raters. In Phase 2, each participant underwent a single assessment performed by two novice raters on the same day in a large sample size. This design was chosen to objectify the reliability of the protocol under conditions corresponding to its first clinical use.

**Figure A1:**
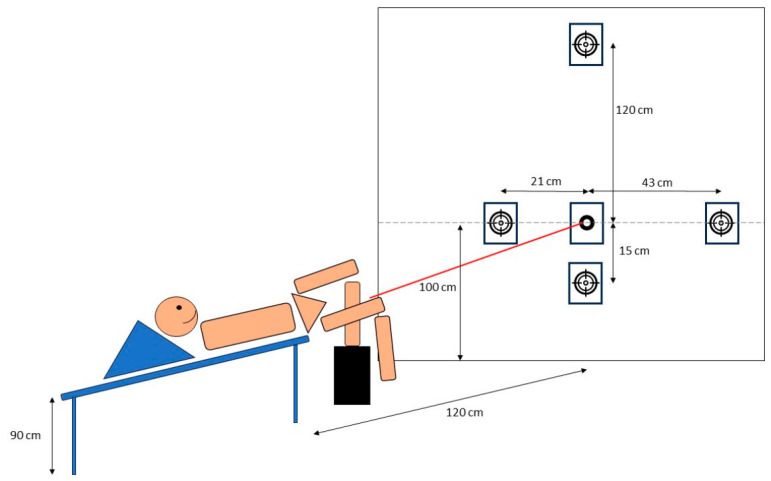
Diagram illustrating the setup for hip assessment (right hip shown on the left side of the figure). All distances were standardized and identical for all participants. The red line represents the laser beam emitted by the laser pointer positioned above the participant’s knee during the assessment.

**Figure A2:**
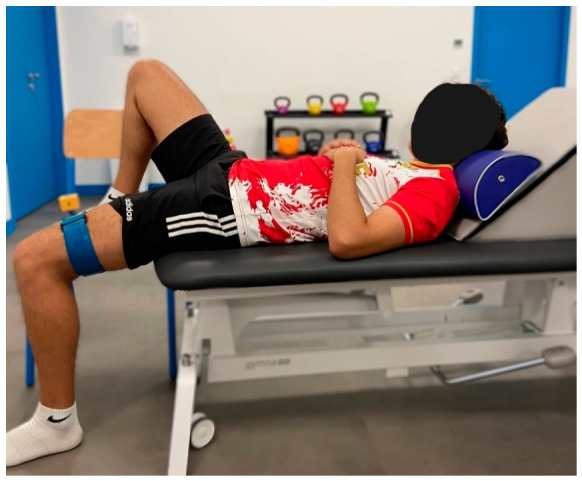
Participant positioning. Side view before the start of the test, with the laser emitter attached to the left thigh.

The participant was positioned supine at the end of the examination table, with both lower limbs facing a wall located 120 cm away (Figure A1). The non-tested limb rested on a chair to stabilise the pelvis and minimise compensatory movements (Figure A2). The tested hip extended beyond the edge of the table to allow a full range of motion, particularly during extension. For all measurements, the knee was kept flexed to prevent muscle fatigue during the session. A full knee extension was required only during the hip extension movement, ensuring that the limb had no contact point or tactile cue with the ground.

**Figure A3:**
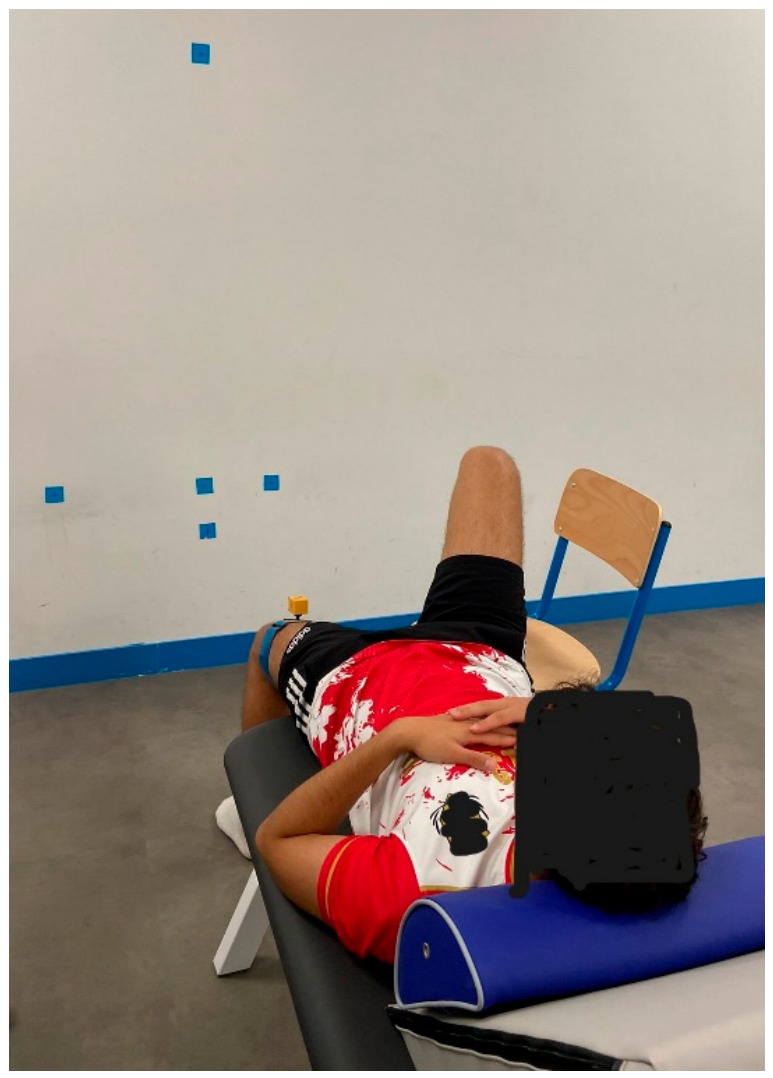
Participant positioning facing the target.

A laser pointer, fixed to the thigh and aligned with the femoral shaft, was used to target five standardized points on the wall: a central target (neutral position), one superior for flexion (120 cm), one inferior for extension (15 cm), and two lateral targets for abduction and adduction (43 cm and 21 cm, respectively) (Figure A1 and Figure A3). These distances corresponded to approximately one-third of the normal physiological hip range of motion (≈38% for flexion, 36% for extension, 44% for abduction, and 33% for adduction), ensuring consistent target positioning while limiting pelvic compensations. The participant’s head was slightly elevated to optimize visibility of the targets.

**Figure A4:**
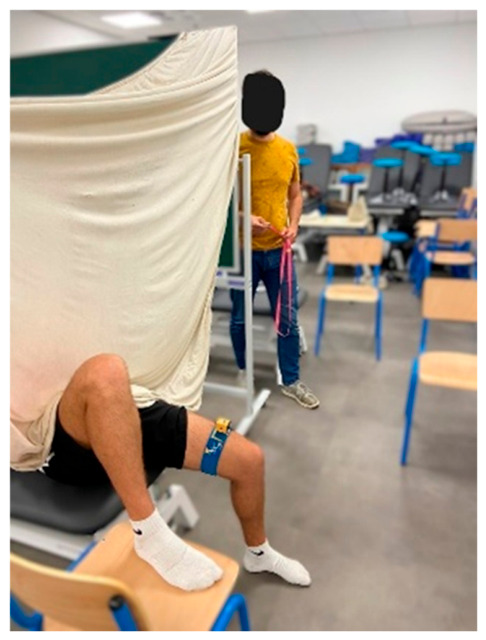
Removable partition and blinding setup. The rater (yellow T-shirt) was blinded to the participant’s identity by the removable partition and was able to take measurements without seeing the upper body. The participant’s lower limbs extended beyond the partition, allowing target aiming without visual feedback.

**Figure A5:**
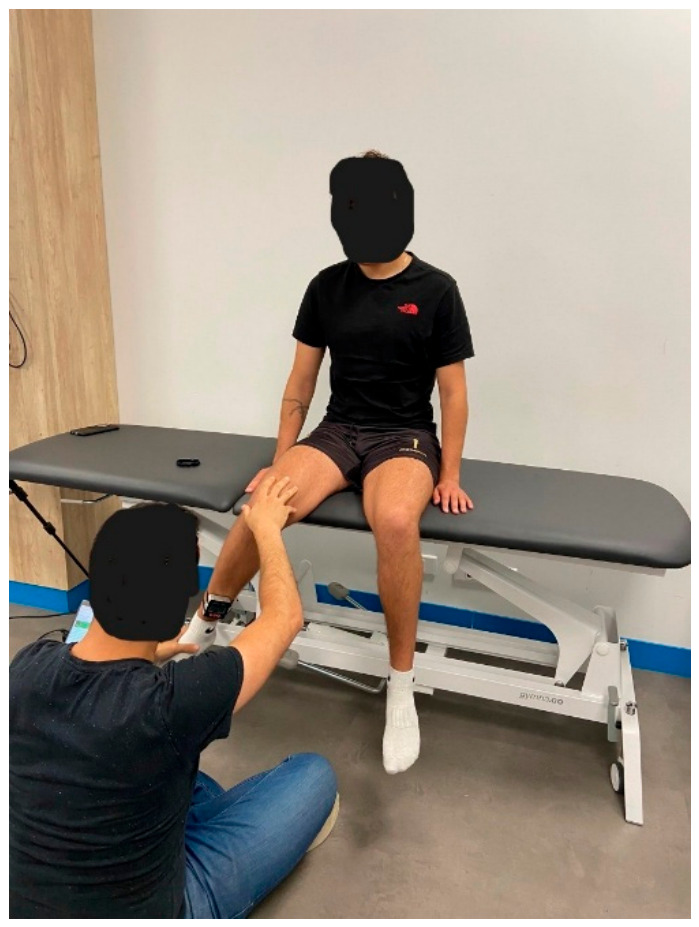
Testing position for assessing joint position error during hip rotations (medial rotation of the right hip shown in the photo).

Two symmetrical setups were installed in each testing room, allowing both hips to be assessed without readjusting the equipment. An opaque curtain combined with a removable partition separated the primary rater from the participant, ensuring blinding (Figure A4). Positioned next to the participant, the co-rater monitored compensatory movements and delivered standardized instructions without providing any feedback, thereby maintaining experimental blinding. The assessment of medial and lateral hip rotations was performed in a seated position, with the hips and knees flexed at 90°. The participant maintained an upright trunk, with both hands resting on the table to stabilise the pelvis. The rater was positioned in front of the participant to guide the movement and ensure the absence of compensatory motions (Figure A5). To prevent any anchoring or expectation bias, raters were systematically blinded to all previous measurements: (i) within a session, the co-rater recorded the values on a separate datasheet not visible to the primary rater; (ii) between Session 1 and Session 2, the rater performing Session 2 had no access to the Session 1 values of the same participant; and (iii) in inter-rater assessments, neither rater had access to the other rater’s values. All data were entered into a secure digital database and unblinded only at the end of data collection.

#### Rater Training and Standardization

All raters (one experienced and three novice raters) participated in a single 2 h standardized training session covering all testing positions, procedures, instrumentation (laser pointer alignment and inclinometer placement), and standardized verbal instructions. Following this training, each rater performed four pilot assessments on volunteers external to the study sample in order to stabilize the procedure and achieve an operator reliability plateau prior to data collection. A written standardized operating procedure was followed by all raters throughout the study [22,23].

### 2.4. Testing Procedure

Each session was conducted individually in a controlled environment to ensure standardisation and participant blinding. The evaluation followed four standardized steps for each leg: (1) Preparation: Verification of eligibility criteria, equipment setup, and delivery of standardized instructions by the co-rater. Participants were instructed to minimize verbal communication to preserve blinding. (2) Flexion, extension, abduction, and adduction measurements: The participant began with the foot placed flat on the floor to calibrate the laser orientation, then aimed successively at the central target and at the designated target. After returning to the neutral position, the participant closed their eyes and reproduced the initial position. An opaque curtain was drawn in front of the participant to prevent visualization of the target and to maintain blinding for both the rater and the participant. (3) Medial and lateral rotation measurements: The rater positioned the hip at 30° of lateral rotation or 15° of medial rotation (target values) and asked the participant to hold the position through an active contraction. The participant was then instructed to memorise the position and reproduce it with their eyes closed. The reproduced angle was measured using a digital inclinometer attached to the anterior tibial crest. (4) End of session: Removal of the equipment and participant release. Several procedural choices were implemented to minimize potential learning and fatigue effects: (i) one standardized familiarization trial was performed for each movement direction prior to the recorded measurements; (ii) the knee remained flexed throughout most assessments to limit muscular fatigue; (iii) the randomized order of movement directions ensured an equal distribution of any residual learning or fatigue effects across conditions; and (iv) the duration of each session was limited to a maximum of 15 min.

### 2.5. Randomisation and Repetitions

The order of movement direction (flexion, extension, abduction, and adduction) and the tested hip side were computer-randomised and remained identical across both sessions. Each movement direction was repeated three times, with a 15 s rest period between repetitions and movements. A 3 min rest period was implemented when changing laterality and prior to the assessment of medial and lateral rotations. For rotational movements, the order of rotation type (medial or lateral) and tested sides was similarly randomised. In addition, a standardized 10 min seated rest period was implemented between reassessments during room transitions.

### 2.6. Instruments

Proprioceptive assessment was performed using an adjustable laser pointer (Motion Guidance^®^, Motion Guidance Laser, LLC, Denver, CO, USA) attached to the thigh with a circular strap positioned 5 cm above the superior border of the patella. For the evaluation of medial and lateral rotations, a digital inclinometer (Hancaner, Shenzhen, China).

### 2.7. Statistical Analysis

#### 2.7.1. Sample Size Calculation

For reliability analyses, the required sample size was calculated in accordance with COSMIN recommendations [26]. Assuming an expected ICC of ρ_1_ = 0.70 (“good” reliability) tested against a null hypothesis of ρ_0_ = 0.50 (“moderate” reliability), with k = 2 ratings per participant, α = 0.05, and a statistical power of 1 − β = 0.80, the required sample size was estimated at approximately 46–50 participants [27]. For measurement error analyses (SEM, MDC, and Bland–Altman agreement), the COSMIN Risk of Bias Checklist recommends a minimum sample size of *n* ≥ 50. Accordingly, Phase 2 included 57 participants, ensuring acceptable methodological quality for both reliability and measurement error analyses. Phase 1 included 17 participants as an exploratory reliability sub-analysis intended to provide preliminary ICC estimates prior to confirmatory testing in Phase 2 [27,28]. Intra-session analyses additionally included k = 3 repeated measurements per participant, further reducing the confidence intervals of intra-session ICC estimates [27]. For the intra-session analysis, reliability was evaluated using ICC(3,1) applied to the three repetitions performed within a single session, with analyses conducted separately for Session 1 and Session 2. Measurement error was quantified using the standard error of measurement (SEM) and the minimal detectable change (MDC), defined respectively as the absolute precision of the measurement and the minimum amount of change needed to exceed measurement noise.

#### 2.7.2. For Preliminary Reference Values

Preliminary reference values were derived by first computing, for each participant and each movement direction, the mean repositioning error from the available trials (12 trials = 3 repetitions × 2 sides × 2 raters; 6 trials when data from only one rater were available). Descriptive statistics (mean, standard deviation, 95% confidence interval, median, first and third quartiles, interquartile range, skewness, and Shapiro–Wilk test for normality) were then computed across the *n* = 57 participants. This participant-level approach preserves the independence-of-observations assumption and reflects inter-individual variability only, rather than pooling intra-trial.

#### 2.7.3. Descriptive Statistics

Descriptive statistics included means, standard deviations, and 95% confidence intervals (95% CI) for all recorded variables. Analyses were performed using JASP software (version 0.17.2.1; JASP Team, University of Amsterdam, Amsterdam, The Netherlands). The normality of data distributions was assessed using the Shapiro–Wilk test and visual inspection of histograms and density plots. No significant differences were found between right and left limb measurements (*p* > 0.05), supporting the decision to perform the main analyses on the right limb only. In total, 336 measurements were obtained for each movement direction (flexion, extension, abduction, adduction, medial rotation, and lateral rotation), forming the preliminary reference values set for the healthy population. Descriptive data are presented in tables and violin plots to illustrate the distribution, mean, 95% CI, and interindividual variability for each movement direction.

#### 2.7.4. Reliability Analysis

Three distinct types of reliability were evaluated, in line with COSMIN terminology [22]: (i) intra-session reliability (consistency of three repeated measurements within the same session, ICC(3,1); (ii) test–retest reliability (stability of measurements performed by the same rater across two sessions separated by 24 h, ICC(3,1); and (iii) inter-rater reliability (agreement between two raters assessing the same participants, ICC(2,1). All ICCs were computed using two-way random-effects models with absolute agreement, following Koo and Li [28]. All ICC calculations were performed using JASP software. For inter-rater analyses, the option “a different rater (randomly selected)” was selected, while for intra-rater analyses, the option “the same set of randomly selected raters/tests” was used, corresponding to the same rater performing multiple repetitions on the same participant. When three repeated measures were performed within the same session (*k* = 3), ICC(3,1) values were calculated based on the mean of the three trials, representing average performance rather than the variability of a single attempt. ICCs were reported with their 95% confidence intervals (95% CI) and interpreted as follows: poor reliability (ICC < 0.50 🔴), moderate reliability (0.50 ≤ ICC < 0.70 🟡), and good reliability (ICC ≥ 0.70 🟢).

The standard error of measurement (SEM) was calculated using the following formula:

$$SEM=SD\times \sqrt{1-ICC}$$

where *SD* represents the standard deviation of the measurements.

The smallest detectable change at the 95% confidence level (*MDC*_95_%) was estimated using the following formula:

$$MDC_{95}=SEM\times 1.96\times \sqrt{2}$$

to determine the *MDC* beyond measurement error. To improve comparability across movement directions, *SEM* and *MDC* values were normalized to the target amplitude of each movement and expressed as percentages.

$$SEM\%=\frac{SEM}{\mathrm{Target}\;\mathrm{amplitude}}\times 100\;MDC\%=\frac{MDC_{95}}{\mathrm{Target}\;\mathrm{amplitude}}\times 100$$

The target amplitudes were defined as follows: flexion = 120 cm, abduction = 43 cm, extension = 15 cm, adduction = 21 cm, medial rotation = 15°, and lateral rotation = 30°. Although no universal thresholds have been established, reference values from the literature were used to guide clinical interpretation [19]: *SEM%* ≤ 10% 🟢 = excellent precision; 11–20% 🟡 = acceptable; >20% 🔴 = low precision; *MDC%* ≤ 15% 🟢 = excellent sensitivity to change; 16–30% 🟡 = moderate; >30% 🔴 = low [20]. These thresholds should be considered contextual clinical benchmarks rather than normative standards. Finally, inter-rater agreement was examined using the Bland–Altman method, which allowed estimation of the mean bias, the standard deviation of the differences, and the 95% limits of agreement (95% CI). The corresponding graphical Bland–Altman plots, with bias, limits of agreement, and their 95% confidence intervals, are provided in the Supplementary Materials.

## 3. Results

For the intra- and inter-rater reliability analyses, a total of 17 participants were included in the study after verification of the inclusion criteria. For the preliminary reference values, data from 57 participants were included in the analyses. Participant demographic characteristics are presented in Table 1.

### 3.1. Phase 1—Intra-Rater Reliability (n = 17)

Detailed results are presented in Table 2. Overall, ICC values ranged from low to moderate across most movement directions, except for flexion and medial rotation, which showed values above 0.60. Intra-rater reliability was consistently higher among experienced raters than novice raters, except for adduction.

Measurement error analysis revealed low to moderate SEM% values for most movements, particularly among experienced raters. The smallest relative errors were observed for medial rotation (4.3%), followed by lateral rotation (6.8%), flexion (8.6%), and abduction (10.1%). Larger errors were found for extension (15.3%) and especially for adduction (16.8%).

Novice raters generally showed higher SEM% values, indicating greater intra-rater variability. Regarding MDC%, most values exceeded 20%, except for lateral rotation in novice raters (10.4%) and for flexion and rotational movements in experienced raters (ranging from 18.9% to 23.9%). The highest MDC% values were observed for adduction (46.5%) and extension (42.5%) among experienced raters, and for adduction (76.4%) and extension (60.5%) among novice raters, indicating low sensitivity to change for these directions.

### 3.2. Phase 2—Intra-Session Reliability (n = 57)

An intra-session reliability analysis was conducted using the three repetitions performed during Session 1 and the three repetitions from Session 2 in the 57 participants who also took part in the inter-rater reliability protocol (Table 3). Measurement stability appeared higher in Session 1 compared to Session 2, except for abduction, extension, and medial rotation.

Overall, SEM% values were low, indicating good measurement precision for most movement directions, particularly for lateral rotation (5.0–5.2%), abduction (8.1–8.7%), and flexion (10.7%). However, MDC% values remained relatively high for most movements, suggesting that a clinically meaningful change within a single session must be substantial to exceed the inherent measurement error of the test. Lateral rotation stood out for its good precision and sensitivity to change, supporting its potential use in clinical practice.

### 3.3. Phase 1—Inter-Rater Reliability (n = 17)

Inter-rater reliability measurements between experienced and novice raters for testing Sessions 1 and 2 are presented in Table A1. Inter-rater analyses showed a consistent improvement in agreement coefficients during Session 2 compared with Session 1. Despite this improvement, ICC values remained generally low, except for medial rotation (ICC = 0.78; 95% CI [0.50; 0.91]), as well as for abduction and extension (ICC = 0.61 and 0.54, respectively) in Session 2.

Conversely, measurements for the other movement directions demonstrated limited reliability, indicating substantial inter-rater variability. SEM% values observed in Session 2 were generally lower, reflecting better measurement precision, particularly for medial rotation (5.1%), lateral rotation (3.8%), and abduction (7.5%). These directions were also associated with low to moderate MDC% values, suggesting that changes detected in these movements are more likely to be clinically interpretable. The Bland–Altman plots and agreement statistics are presented in Table A2 and the Supplementary Materials.

### 3.4. Phase 2—Inter-Rater Reliability (n = 57)

Inter-rater reliability measurements between novice raters for the 57 participants are reported in Table 4a, with limits of agreement presented in Table 4b. Inter-rater analyses performed on this sample showed generally low ICC values, reflecting substantial variability between raters. Abduction demonstrated the highest ICC (ICC = 0.45; 95% CI [0.22; 0.63]), while the other movement directions displayed low to very low coefficients.

Conversely, SEM% values were low to moderate, with particularly satisfactory results for abduction (4.4%), lateral rotation (8.1%), and flexion (8.3%), indicating good measurement precision despite the overall weak concordance. MDC% values were higher, suggesting that a clinically meaningful change would require relatively large variations, especially for rotational movements. Bland–Altman analyses revealed small mean biases, indicating no systematic bias, but wide limits of agreement, confirming notable inter-rater dispersion (Table 4b and the Supplementary Materials).

### 3.5. Phase 2—Preliminary Reference Values in a Healthy Population (n = 57)

**Figure 2:**
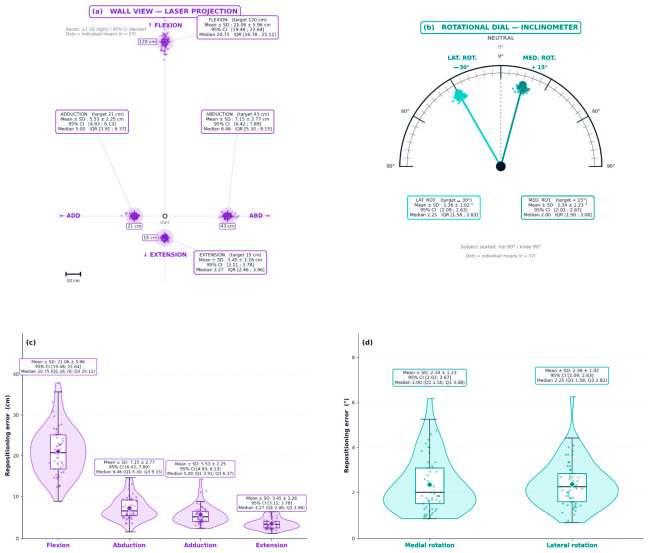
Preliminary reference values for the active hip joint-position-reproduction error in healthy adults (Phase 2; *n* = 57). (**a**) Wall view of the laser projection (sagittal and frontal planes): the four anterior targets (flexion 120 cm, extension 15 cm, abduction 43 cm, adduction 21 cm) are drawn to scale; light shading = ±1 SD band, denser shading = 95% CI band, purple dots = individual participant means. (**b**) Rotational dial of the inclinometer (transverse plane, hip/knee at 90°): needles point to the medial-rotation (+15°) and lateral-rotation (−30°) targets, with individual participant means scattered around each target. (**c**) Violin plots, embedded boxplots (median, Q1–Q3, 1.5 × IQR whiskers), jittered individual means and arithmetic means (◆) for the four laser-based linear movements (cm). (**d**) Same conventions as (**c**) for the two inclinometer-based rotational movements (°). Each data point represents one participant’s mean repositioning error computed from 12 trials (3 repetitions × 2 sides × 2 raters), or from 6 trials for two participants for whom data from only one rater were available. Abbreviations: CI—confidence interval; IQR—interquartile range; Q1—first quartile; Q3—third quartile; SD—standard deviation.

Preliminary reference values for the healthy population are presented in Figure 2. A total of 336 measurements per movement direction were analysed to establish reference values for hip joint position reproduction errors in a healthy sample. The mean errors observed were 21.06 ± 5.96 cm (95% CI [19.48; 22.64]) for flexion, 7.15 ± 2.77 cm (95% CI [6.42; 7.89]) for abduction, 5.53 ± 2.25 cm (95% CI [4.93; 6.13]) for adduction, and 3.45 ± 1.26 cm (95% CI [3.11; 3.78]) for extension. For rotational movements, errors were substantially smaller, with 2.34 ± 1.23° (95% CI [2.02; 2.67]) for medial rotation and 2.36 ± 1.02° (95% CI [2.09; 2.63]) for lateral rotation. Overall, data dispersion was greater for movements in the frontal and sagittal planes than for rotational movements.

## 4. Discussion

The primary objective of this two-phase study was to evaluate the intra-rater, inter-rater, and test–retest reliability of a standardized laser- and inclinometer-based protocol for measuring active hip joint position error and to establish preliminary reference values in healthy adults. Intra-rater reliability ranged from poor to moderate across movement directions (ICC = 0.00–0.64), with consistently higher values for experienced raters than for novice raters and a clear trend toward better reliability for movements involving small amplitudes and proximal control. Inter-rater reliability was overall low and heterogeneous across directions (ICC = 0.01–0.78), but showed a marked improvement between Session 1 and Session 2, suggesting a learning effect inherent to the proprioceptive task and to rater–participant interaction. Among all assessed directions, medial and lateral rotations consistently appeared to be the most clinically informative: medial rotation demonstrated the highest inter-rater reliability (ICC = 0.78; 95% CI [0.50–0.91]), and both rotational movements showed the lowest SEM% values (3–6%, within clinically acceptable limits), together with the lowest normative repositioning errors (2.34° ± 1.23° for medial rotation; 2.36° ± 1.02° for lateral rotation). Preliminary reference values showed larger reproduction errors in the sagittal and frontal planes and markedly smaller errors for rotations.

### 4.1. Intra-Rater and Intra-Session Reliability

These findings may reflect the sensitivity of the ICC to between-subject variance, the limited number of trials, and the small sample size [28]. Rater expertise is also likely to play a role, consistent with evidence on lower-limb JPS reproducibility [29]. However, much of the variability probably stems from the participants themselves, who were novices in a demanding proprioceptive task requiring simultaneous motor command, motor control, interhemispheric communication, and working memory. Such complexity naturally produces fluctuating performance that may improve with practice. The better reliability observed in Session 2 may therefore indicate progressive sensorimotor adaptation or optimization of motor strategies across trials [30], suggesting that a single familiarization trial per direction was insufficient to fully stabilise performance.

### 4.2. Reliability Across Movement Directions: Are Rotations a Key Factor?

Medial rotation, in particular, demonstrated higher ICC values than those reported by Takahashi et al. (2024) [31]. Several factors may account for this enhanced reproducibility: (1) direct tension of the anterior and posterior capsule and the labrum, structures rich in mechanoreceptors that provide dense proprioceptive feedback [32,33]; (2) reduced susceptibility to lumbar and pelvic compensations compared with movements performed in the supine position; (3) targeted activation of deep hip rotators, many with proximal attachments on the lower limb and a high density of muscle spindles (Ia and II afferents), enabling fine angular discrimination [34]; and (4) the nature of the measurement tools and tasks: inclinometer-based angular measures are less prone to multisegmental compensations than laser-derived linear errors, which are amplified by target distance. In addition, large-amplitude tasks (e.g., hip flexion or abduction) involve multiple segments and therefore generate more variability than rotational movements.

### 4.3. Overall Reflection on the Contrast Between ICC, SEM%, and MDC%

The combination of a low SEM% and a high MDC% suggests good measurement precision but limited trial-to-trial stability [35], a pattern frequently reported in proprioceptive assessments and reflecting known methodological limitations of joint position sense (JPS) and threshold to detect passive motion (TTDPM) tests [29,36]. This dissociation between average precision and consistency likely reflects four converging sources of variability: (i) participant-level stochastic fluctuations along the neuromuscular pathway, from action potential generation to neuromuscular transmission and muscle fiber activation [25,37]; (ii) task-level multisegmental compensations during large-amplitude movements (flexion, abduction), which amplify laser-derived linear errors [38]; (iii) rater-level experience effects, with experienced raters consistently outperforming novice raters across most movement directions (see Table 2 and Table 3), consistent with previous lower-limb JPS data [29]; and (iv) tool-level robustness, with inclinometer-based angular measures (rotations) being less affected than laser-derived linear errors amplified by target distance. In our joint position reproduction protocol, the trial-to-trial variability likely reflects this fundamental sensorimotor uncertainty, largely independent of the mean task precision.

From a clinical standpoint, these findings suggest that performing a same-session test–retest in rotational positions with an inclinometer, which showed the lowest measurement error, may be the most effective approach to detect changes exceeding the MDC% and to identify potential “good responders”. Considered jointly with their clinical interpretation, the SEM% values observed for rotational movements fall well within the threshold considered clinically acceptable for proprioceptive assessment tools (SEM% < 10% [24], identifying these directions as the most promising candidates for further clinimetric validation. Conversely, the MDC% values observed for sagittal and frontal-plane movements (frequently >30%) exceed the threshold of clinically interpretable change [24], suggesting that these directions, in their current measurement form, may not be suitable for detecting individual within-subject changes in clinical practice.

### 4.4. Preliminary Reference Values (n = 57)

The preliminary reference values obtained here provide the first reference benchmarks for hip joint position reproduction error in a healthy population. The hierarchy of variability, greater in the sagittal and frontal planes, smaller in rotational movements, likely reflects the combined contribution of pelvic compensations, the density of capsulolabral and muscle spindle inputs, and the measurement modality itself. This hierarchy of stability (rotations > small-amplitude movements > large-amplitude movements) appears clinically meaningful. Rotational tasks may offer a reliable entry point for assessment, while more complex movements can be used to test or train higher-level proprioceptive demands. These preliminary reference values may provide an initial methodological basis for future research. However, any clinical application, such as patient stratification, rehabilitation monitoring, or evaluation of therapeutic progression, would require further validation studies.

### 4.5. Strengths and Limitations

This study has several strengths, including strict adherence to COSMIN and GRRAS recommendations, the use of simple, accessible, and reproducible tools (laser pointer and inclinometer), and the inclusion of raters with different levels of experience alongside a healthy normative sample, providing novel insights into hip proprioception. Several limitations must, however, be acknowledged: the small Phase 1 sample, which resulted in wide confidence intervals; the exclusive use of active measurements, producing a composite assessment of proprioceptive and motor functions rather than isolated proprioception [29,39]; the short familiarization period, which may have increased inter-session variability; and the inclusion of healthy participants only, limiting clinical generalizability. It should also be noted that, although proprioceptive retraining may improve joint position sense, current evidence does not conclusively demonstrate that such gains translate into meaningful improvements in quality of life or patient-reported outcomes [40]. Finally, laser-based linear measurements (cm) and inclinometer-based angular measurements (°) being dimensionally distinct, absolute SEM and MDC values are not directly comparable across modalities; cross-direction comparisons were therefore restricted to the normalized SEM% and MDC% [24].

## 5. Conclusions

This two-phase study aimed to evaluate the intra-rater, inter-rater, and test–retest reliability of a standardized laser- and inclinometer-based protocol for measuring active hip JPS, and to establish preliminary reference values in a healthy population. The results demonstrated satisfactory measurement precision (low SEM%) but overall low to moderate relative reliability (ICC = 0.00–0.78), influenced by rater experience, movement direction, and the inherent fluctuations of sensorimotor control. Medial and lateral rotations showed the most favourable trade-off between relative reliability and absolute measurement error (SEM% = 3–6%, within clinically acceptable limits), supporting their potential clinical relevance for individual-level hip proprioceptive monitoring. The preliminary reference values obtained provide an initial reference for the clinical interpretation of active hip JPS performance and lay the groundwork for future studies in symptomatic populations.

## Figures and Tables

**Table 1:** Participant characteristics, Phase 1 (intra- and inter-rater reliability, *n* = 17) and Phase 2 (inter-rater reliability and preliminary reference values (*n* = 57)).

Variable	Phase 1 (*n* = 17) Mean ± SD	Phase 2 (*n* = 57) Mean ± SD
**Age (years)**	38.1 ± 15.8	31.1 ± 11.8
**Height (cm)**	166.5 ± 5.3	172.1 ± 8.3
**Weight (kg)**	67.8 ± 10.7	71.6 ± 14.4
**Sex (M/F)**	4 (25%)/13 (75%)	28 (48%)/29 (52%)
**Right dominance**	15 (88%)	51 (91%)
**Occupation**	Physiotherapist: 6 (38%) Administrative assistant: 3 (19%) Teacher (physical education): 1 (6%) Nurse: 1 (6%) Accountant: 1 (6%) Driver/mechanic: 1 (6%) Housekeeper: 1 (6%) Dental student: 1 (6%) Unemployed: 1 (6%)	Student: 20 (36%) Physiotherapist: 13 (23%) Administrative assistant: 3 (5%) Osteopath: 2 (4%) Other (education, office, manual work): 18 (32%)
**Current sport participation**	No physical activity: 7 (44%) Dance/Pilates: 5 (31%) Strength training/Cycling: 2 (13%) Other: 2 (13%)	No physical activity: 11 (20%) Running: 9 (16%) Strength training/Fitness/CrossFit: 13 (23%) Team sports (hockey, football, rugby, basketball): 6 (11%) Dance/Gymnastics/Pilates: 5 (9%) Combat sports (boxing, taekwondo, CAP): 3 (5%) Other (climbing, athletics, cycling, calisthenics): 9 (16%)
**Level of sport participation**	RS = 9/N = 7	RS = 35/DC = 8/*CR* = 6/*NC* = 7
**Current sport frequency (sessions/week)**	2.9 ± 1.7	2.8 ± 1.6
**Youth sport frequency (sessions/week)**	3.1 ± 1.0	3.0 ± 1.3
**Youth sport duration (years)**	5.6 ± 2.3	6.2 ± 3.1
**Youth sport level (L/CD/CR/CN/CI)**	RS = 5/DC = 6/*NC* = 4/IC = 1	RS = 24/DC = 8/*CR* = 6/*NC* = 13
**Previous injury (any)**	10 (59%)	30 (54%)
**Injury type**	Ankle sprain: 8 (47%); hamstring strain: 2 (12%); fracture: 2 (12%)	Ankle sprain: 20 (36%) Knee ligament injury: 5 (9%) Fracture: 6 (11%) Other injuries: 15 (27%)
**Recent hip condition (<6 months)**	0 (0%)	0 (0%)
**Medication use**	4 (24%)	8 (14%)

In this table, RS refers to recreational sports practice, DC to departmental competition, CR to regional competition, NC to national competition, and IC to international competition. The letters F and M indicate female and male sex, respectively.

**Table 2:** Intra-rater reliability of hip joint position error for experienced raters (a) and novice raters (b), *n* = 17.

(a) Experienced Raters					
Movement	ICC(3,1) [95% CI]	SEM	SEM%	MDC	MDC%
**Flexion (cm)**	0.64 [0.39; 0.80] 🟡	10.37	8.6 🟢	28.74	23.9 🟡
**Abduction (cm)**	0.46 [0.12; 0.67] 🔴	4.33	10.1 🟢	12.01	27.9 🟡
**Extension (cm)**	0.00 [−0.33; 0.34] 🔴	2.30	15.3 🟡	6.37	42.5 🔴
**Adduction (cm)**	0.16 [−0.18; 0.47] 🔴	3.52	16.8 🟡	9.76	46.5 🔴
**Medial rotation (°)**	0.64 [0.40; 0.80] 🟡	1.10	7.3 🟢	3.02	20.1 🟡
**Lateral rotation (°)**	0.21 [−0.13; 0.51] 🔴	2.04	6.8 🟢	5.66	18.9 🟡
(b) Novice Raters					
Movement	ICC(3,1) [95% CI]	SEM	SEM%	MDC	MDC%
**Flexion (cm)**	0.02 [−0.32; 0.35] 🔴	12.89	10.7 🟢	35.72	29.8 🟡
**Abduction (cm)**	0.30 [−0.05; 0.57] 🔴	4.30	10.0 🟢	11.93	27.7 🟡
**Extension (cm)**	0.00 [−0.33; 0.33] 🔴	3.27	21.8 🔴	9.07	60.5 🔴
**Adduction (cm)**	0.22 [−0.12; 0.52] 🔴	5.80	27.6 🔴	16.05	76.4 🔴
**Medial rotation (°)**	0.10 [−0.24; 0.42] 🔴	1.99	13.3 🟡	5.52	36.8 🔴
**Lateral rotation (°)**	0.00 [−0.33; 0.33] 🔴	1.12	3.7 🟢	3.11	10.4 🟢

ICC = Intraclass Correlation Coefficient; SEM = Standard Error of Measurement; MDC = Minimal Detectable Change; 95% CI = 95% Confidence Interval. 🟢 = excellent; 🟡 = acceptable/moderate; 🔴 = low.

**Table 3:** Intra-session reliability (*n* = 57).

(a) Session 1					
Movement	ICC(3,1) [95% CI]	SEM	SEM%	MDC	MDC%
**Flexion (cm)**	0.26 [0.10; 0.43] 🔴	12.84	10.7 🟢	35.60	29.7 🟡
**Abduction (cm)**	0.49 [0.33; 0.63] 🔴	3.75	8.7 🟢	10.40	24.2 🟡
**Extension (cm)**	0.33 [0.17; 0.50] 🔴	1.90	12.7 🟡	5.29	35.3 🔴
**Adduction (cm)**	0.71 [0.60; 0.81] 🟢	2.07	9.9 🟢	5.75	27.4 🟡
**Medial rotation (°)**	0.58 [0.43; 0.70] 🟡	1.71	11.4 🟡	4.74	31.6 🔴
**Lateral rotation (°)**	0.77 [0.67; 0.85] 🟢	1.51	5.0 🟢	4.19	14.0 🟢
(b) Session 2					
Movement	ICC(3,1) [95% CI]	SEM	SEM%	MDC	MDC%
**Flexion (cm)**	0.16 [0.01; 0.34] 🔴	12.85	10.7 🟢	35.62	29.7 🟡
**Abduction (cm)**	0.57 [0.43; 0.70] 🟡	3.47	8.1 🟢	9.62	22.4 🟡
**Extension (cm)**	0.59 [0.45; 0.72] 🟡	1.44	9.6 🟢	3.99	26.6 🟡
**Adduction (cm)**	0.52 [0.37; 0.66] 🟡	2.63	12.5 🟡	7.30	34.8 🔴
**Medial rotation (°)**	0.72 [0.61; 0.82] 🟢	1.51	10.1 🟢	4.18	27.9 🟡
**Lateral rotation (°)**	0.68 [0.57; 0.78] 🟡	1.55	5.2 🟢	4.30	14.3 🟢

ICC = Intraclass Correlation Coefficient; SEM = Standard Error of Measurement; MDC = Minimal Detectable Change; 95% CI = 95% Confidence Interval. 🟢 = excellent; 🟡 = acceptable/moderate; 🔴 = low.

**Table 4:** Inter-rater reliability and agreement between novice raters (*n* = 57) participants.

**(a) Inter-Rater Reliability**					
**Movement**	**ICC(2,1) [95% CI]**	**SEM**	**SEM%**	**MDC**	**MDC%**
**Flexion (cm)**	0.12 [−0.14; 0.36] 🔴	9.96	8.3 🟢	27.61	23.0 🟡
**Abduction (cm)**	0.45 [0.22; 0.63] 🔴	3.17	7.4 🟢	8.80	20.5 🟡
**Extension (cm)**	0.01 [−0.25; 0.27] 🔴	1.69	11.3 🟡	4.68	31.2 🔴
**Adduction (cm)**	0.28 [0.02; 0.50] 🔴	2.82	13.4 🟡	7.81	37.2 🔴
**Medial rotation (°)**	0.13 [−0.10; 0.36] 🔴	2.30	15.3 🟡	6.36	42.4 🔴
**Lateral rotation (°)**	0.18 [−0.07; 0.42] 🔴	2.42	8.1 🟢	6.71	22.4 🟡
**(b) Bland–Altman Analysis Showing Inter-Rater Agreement Limits**					
**Movement**	**Mean Bias [95% CI]**	**SD**	**Lower LOA [95% CI]**	**Upper LOA [95% CI]**	
**Flexion (cm)**	2.07 [−1.76; 5.89]	14.42	−26.19 [−32.77; −19.62]	30.32 [23.75; 36.90]	
**Abduction (cm)**	−0.24 [−1.65; 1.17]	5.32	−10.65 [−13.08; −8.23]	10.18 [7.76; 12.61]	
**Extension (cm)**	−0.33 [−0.99; 0.34]	2.52	−5.26 [−6.41; −4.11]	4.61 [3.46; 5.76]	
**Adduction (cm)**	0.31 [−0.80; 1.42]	4.19	−7.89 [−9.80; −5.98]	8.52 [6.61; 10.43]	
**Medial rotation (°)**	1.69 [0.57; 2.81]	4.21	−6.57 [−8.48; −4.64]	9.94 [8.02; 11.86]	
**Lateral rotation (°)**	0.40 [−1.11; 1.91]	5.69	−10.75 [−13.36; −8.15]	11.57 [8.96; 14.16]	

ICC = Intraclass Correlation Coefficient; SEM = Standard Error of Measurement; MDC = Minimal Detectable Change; SD = Standard Deviation; LOA = Limits of Agreement; 95% CI = 95% Confidence Interval. 🟢 = excellent; 🟡 = acceptable/moderate; 🔴 = low.

## Data Availability

The datasets generated and analyzed during the current study are not publicly available due to institutional and ethical restrictions. However, data may be made available from the corresponding author upon reasonable request, pending ethics approval and compliance with confidentiality requirements.

## Supplementary Materials

The following supporting information can be downloaded at: https://www.mdpi.com/article/10.3390/muscles5020045/s1.

## Appendix A

Additional methodological details, experimental setup, participant positioning, and blinding procedures are provided in Figure A1, Figure A2, Figure A3, Figure A4 and Figure A5. Extended reliability analyses, including inter-rater reliability results (Sessions 1 and 2) and Bland–Altman agreement plots, are presented in Table A1 and Table A2 to support the interpretation of measurement properties and enhance transparency and reproducibility of the findings.

**Table A1:** Inter-rater reliability between Session 1 (a) and Session 2 (b).

(a) Session 1					
Movement	ICC(3,1) [95% CI]	SEM	SEM%	MDC	MDC%
**Flexion (cm)**	0.27 [−0.22; 0.65] 🔴	9.59	8.0 🟢	26.57	22.1 🟡
**Abduction (cm)**	0.01 [−0.45; 0.47] 🔴	5.37	12.5 🟡	14.89	34.6 🔴
**Extension (cm)**	0.45 [−0.01; 0.76] 🔴	1.55	10.3 🟢	4.28	28.5 🟡
**Adduction (cm)**	0.00 [−0.46; 0.47] 🔴	5.66	27.0 🔴	15.68	74.7 🔴
**Medial rotation (°)**	0.40 [−0.07; 0.73] 🔴	1.69	11.3 🟡	4.69	31.3 🔴
**Lateral rotation (°)**	0.19 [−0.15; 0.49] 🔴	0.97	3.2 🟢	2.70	9.0 🟢
(b) Session 2					
Movement	ICC(3,1) [95% CI]	SEM	SEM%	MDC	MDC%
**Flexion (cm)**	0.46 [0.00; 0.76] 🔴	13.57	11.3 🟡	37.62	31.4 🔴
**Abduction (cm)**	0.61 [0.22; 0.84] 🟡	3.21	7.5 🟢	8.90	20.7 🟡
**Extension (cm)**	0.54 [0.11; 0.81] 🟡	1.99	13.3 🟡	5.52	36.8 🔴
**Adduction (cm)**	0.49 [0.04; 0.78] 🔴	3.60	17.1 🟡	9.98	47.5 🔴
**Medial rotation (°)**	0.78 [0.50; 0.91] 🟢	0.77	5.1 🟢	2.15	14.3 🟢
**Lateral rotation (°)**	0.27 [−0.07; 0.55] 🔴	1.13	3.8 🟢	3.14	10.5 🟢

ICC = Intraclass Correlation Coefficient; SEM = Standard Error of Measurement; MDC = Minimal Detectable Change; 95% CI = 95% Confidence Interval. 🟢 = excellent; 🟡 = acceptable/moderate; 🔴 = low.

**Table A2:** Bland–Altman analysis of inter-rater agreement between Session 1 (a) and Session 2 (b).

(a) Session 1				
Movement	Mean bias [95% CI]	SD	Lower LOA [95% CI]	Upper LOA [95% CI]
**Flexion (cm)**	0.92 [−0.22; 0.65]	14.03	−26.58 [−39.16; −14.00]	28.42 [15.84; 40.10]
**Abduction (cm)**	0.45 [−3.76; 4.66]	8.19	−15.61 [−22.95; −8.26]	16.50 [9.16; 23.85]
**Extension (cm)**	0.63 [−0.49; 1.74]	2.17	−3.62 [−5.57; −1.68]	4.88 [2.93; 6.82]
**Adduction (cm)**	1.58 [−2.62; 5.78]	8.17	−14.43 [−21.75; −7.10]	17.59 [10.27; 24.92]
**Medial rotation (°)**	−0.61 [−2.14; 0.93]	2.98	−6.45 [−9.13; −3.77]	5.24 [2.57; 7.91]
**Lateral rotation (°)**	1.10 [−0.04; 2.23]	2.21	−3.23 [−5.22; −1.25]	5.43 [3.44; 7.41]
(b) Session 2				
Movement	Mean bias [95% CI]	SD	Lower LOA [95% CI]	Upper LOA [95% CI]
**Flexion (cm)**	1.30 [−8.93; 11.53]	19.89	−37.69 [−55.53; −19.86]	40.29 [22.45; 58.13]
**Abduction (cm)**	1.90 [−0.62; 4.40]	4.90	−7.70 [−12.09; −3.31]	11.50 [7.11; 15.89]
**Extension (cm)**	−1.08 [−2.47; −0.30]	2.69	−6.36 [−8.78; −3.95]	4.20 [1.78; 6.62]
**Adduction (cm)**	0.69 [−2.00; 3.38]	5.24	−9.58 [−14.28; −4.88]	10.96 [6.26; 15.65]
**Medial rotation (°)**	0.16 [−1.07; 1.38]	2.39	−4.52 [−6.66; −2.38]	4.83 [2.69; 6.97]
**Lateral rotation (°)**	0.43 [−0.37; 1.23]	1.55	−2.61 [−4.01; −1.22]	3.48 [2.08; 4.87]

SD = Standard Deviation; LOA = Limits of Agreement; 95% CI = 95% Confidence Interval.
