# Supplementary material for: Intra-Rater, Inter-Rater, and Test–Retest Reliability of a Laser- and Inclinometer-Based Hip Joint Position Sense Test in Healthy Adults: A Two-Phase Study with Preliminary Reference Values
Source: Muscles. 2026 Jun 19;5(2):45. doi: 10.3390/muscles5020045 (PMC13304680; doi:10.3390/muscles5020045)
Supplement: Supplementary file 1 [file muscles-05-00045-s001.zip › muscles-4284852-supplementary.pdf]

Supplementary Materials

Supplementary Materials — Inter-rater agreement, Phase 1 — Session 1 (n = 17)

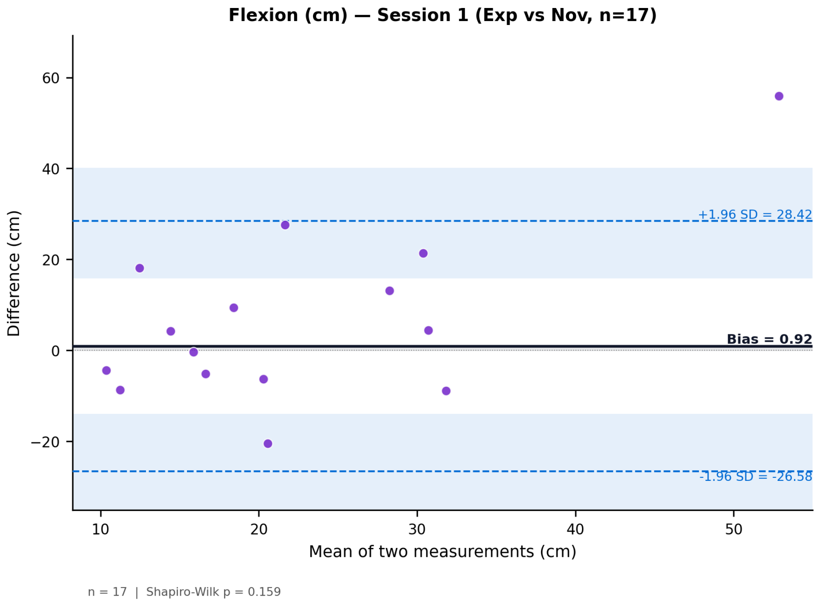

Figure S1. Bland–Altman plot of inter-rater agreement between the experienced and novice raters for flexion — Session 1 (n = 17). Mean bias = 0.92 cm [95% CI -0.22 ; 0.65]; lower LoA = -26.58 cm [-39.16 ; -14.00]; upper LoA = 28.42 cm [15.84 ; 40.10]; SD of differences = 14.03 cm.

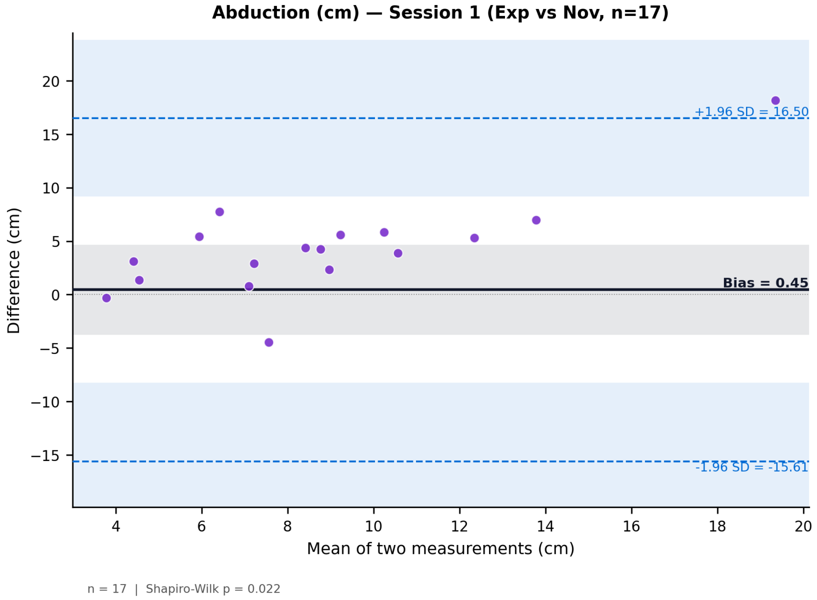

Figure S2. Bland–Altman plot of inter-rater agreement between the experienced and novice raters for abduction — Session 1 (n = 17). Mean bias = 0.45 cm [95% CI -3.76 ; 4.66]; lower LoA = -15.61 cm [-22.95 ; -8.26]; upper LoA = 16.50 cm [9.16 ; 23.85]; SD of differences = 8.19 cm.

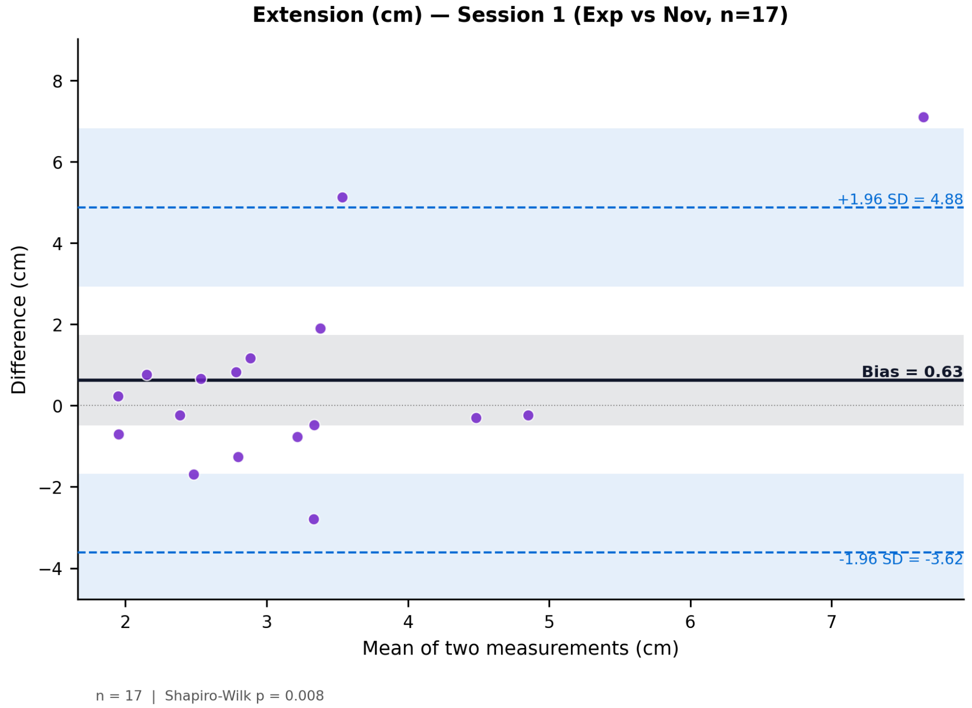

Figure S3. Bland–Altman plot of inter-rater agreement between the experienced and novice raters for extension — Session 1 (n = 17). Mean bias = 0.63 cm [95% CI -0.49 ; 1.74]; lower LoA = -3.62 cm [-5.57 ; -1.68]; upper LoA = 4.88 cm [2.93 ; 6.82]; SD of differences = 2.17 cm.

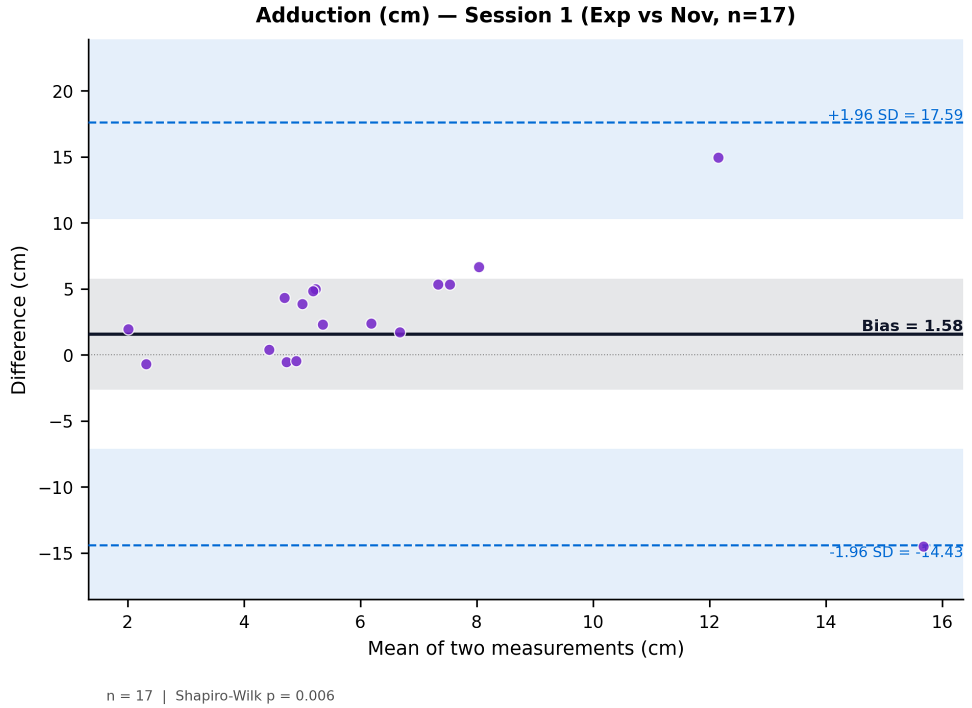

Figure S4. Bland–Altman plot of inter-rater agreement between the experienced and novice raters for adduction — Session 1 (n = 17). Mean bias = 1.58 cm [95% CI -2.62 ; 5.78]; lower LoA = -14.43 cm [-21.75 ; -7.10]; upper LoA = 17.59 cm [10.27 ; 24.92]; SD of differences = 8.17 cm.

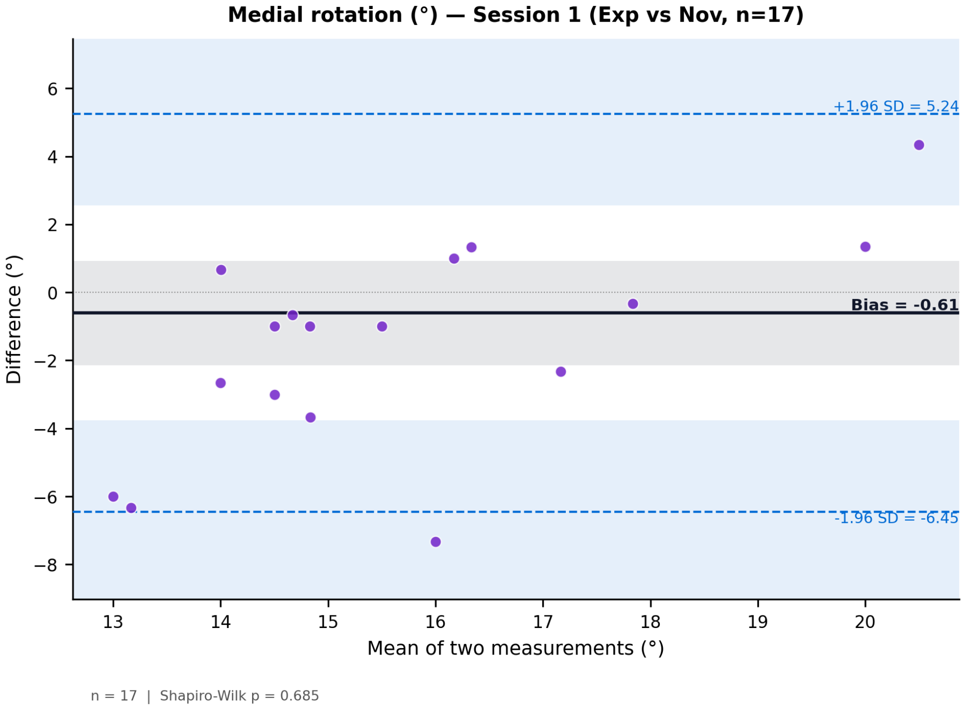

Figure S5. Bland–Altman plot of inter-rater agreement between the experienced and novice raters for medial rotation — Session 1 (n = 17). Mean bias = -0.61 ° [95% CI -2.14 ; 0.93]; lower LoA = -6.45 ° [-9.13 ; -3.77]; upper LoA = 5.24 ° [2.56 ; 7.91]; SD of differences = 2.98 °.

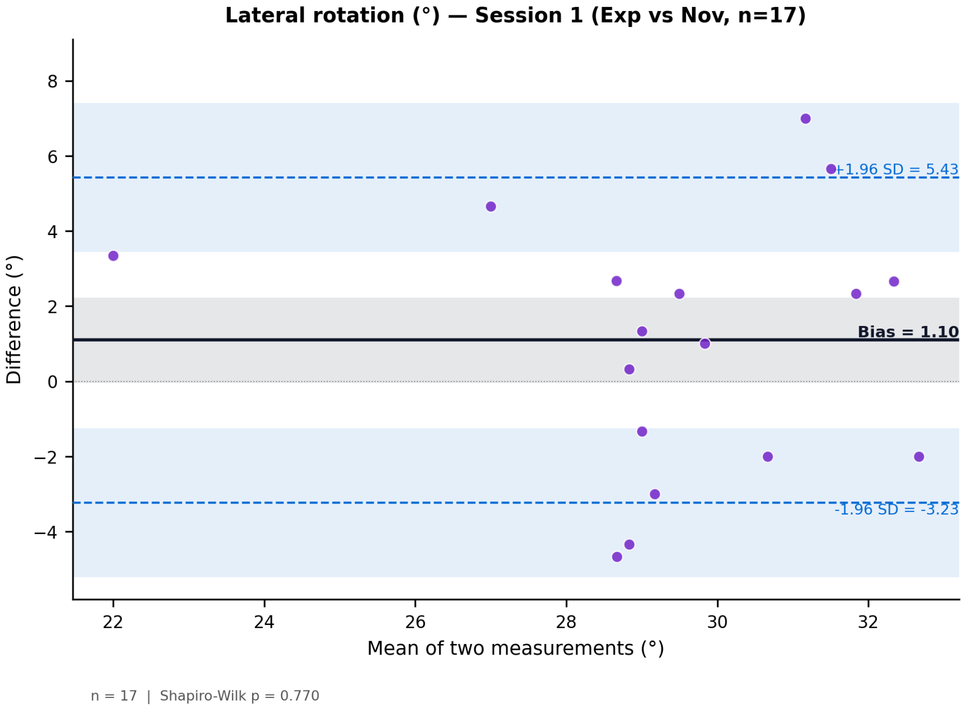

Figure S6. Bland–Altman plot of inter-rater agreement between the experienced and novice raters for lateral rotation — Session 1 (n = 17). Mean bias = 1.10 ° [95% CI -0.04 ; 2.23]; lower LoA = -3.23 ° [-5.22 ; -1.25]; upper LoA = 5.43 ° [3.44 ; 7.41]; SD of differences = 2.21 °.

Supplementary Materials — Inter-rater agreement, Phase 1 — Session 2 (n = 17)

Figure S7 — Flexion (cm)

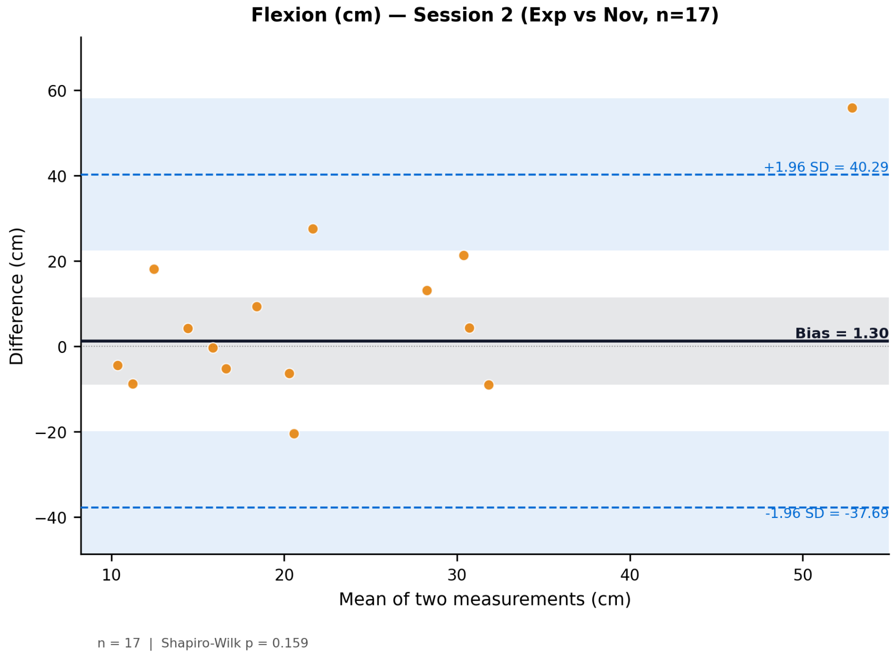

Figure S7. Bland–Altman plot of inter-rater agreement between the experienced and novice raters for flexion — Session 2 (n = 17). Mean bias = 1.30 cm [95% CI -8.93 ; 11.53]; lower LoA = -37.69 cm [-55.53 ; -19.86]; upper LoA = 40.29 cm [22.45 ; 58.13]; SD of differences = 19.89 cm.

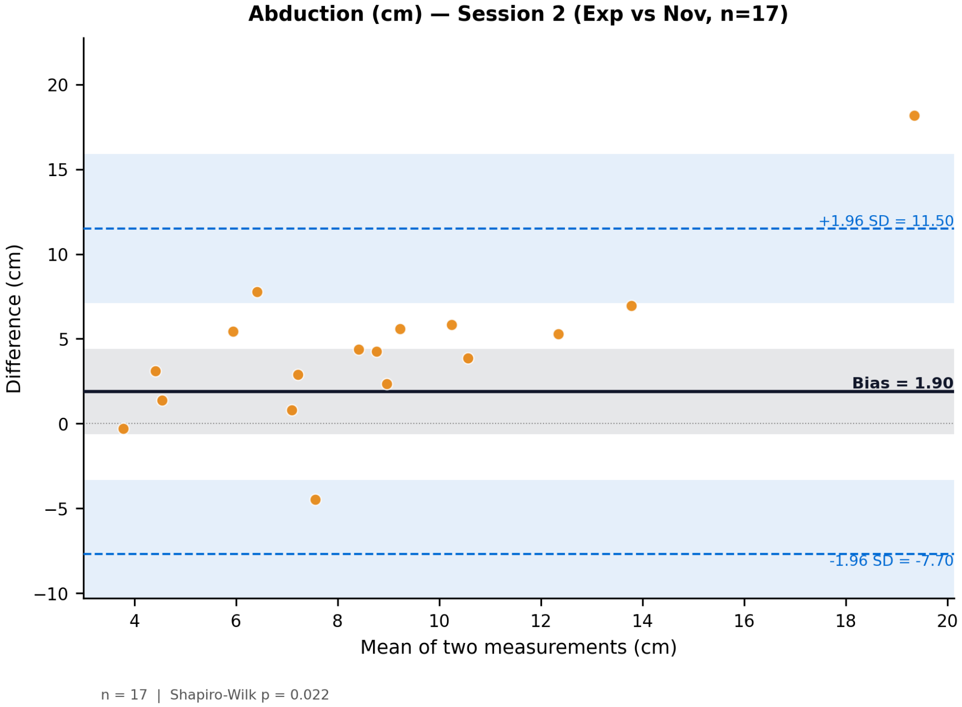

Figure S8. Bland–Altman plot of inter-rater agreement between the experienced and novice raters for abduction — Session 2 (n = 17). Mean bias = 1.90 cm [95% CI -0.62 ; 4.40]; lower LoA = -7.70 cm [-12.09 ; -3.31]; upper LoA = 11.50 cm [7.11 ; 15.89]; SD of differences = 4.90 cm.

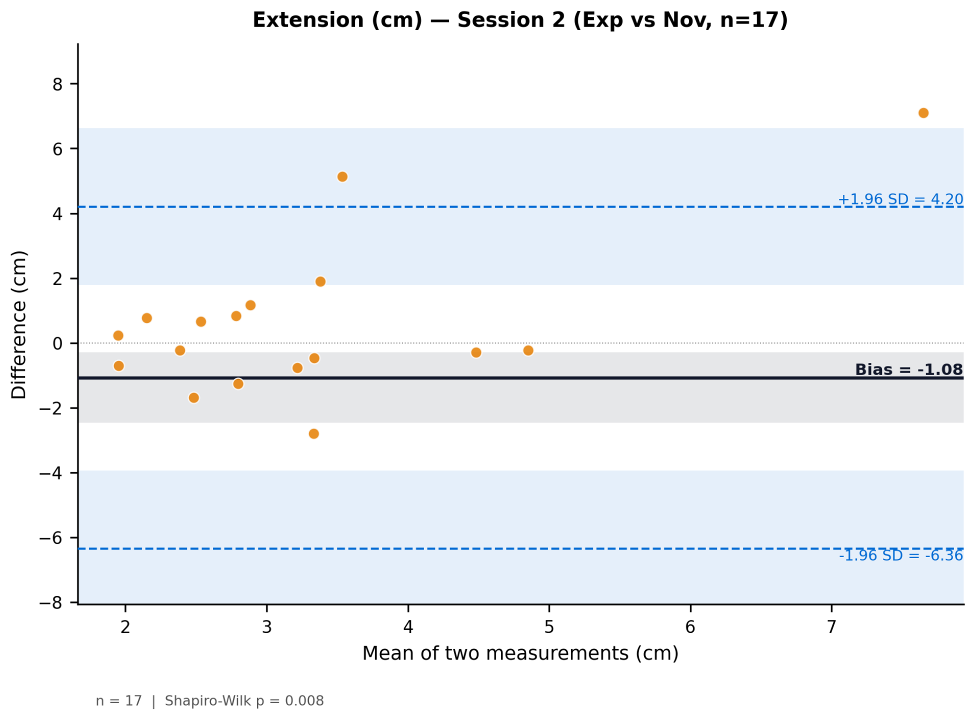

Figure S9. Bland–Altman plot of inter-rater agreement between the experienced and novice raters for extension — Session 2 (n = 17). Mean bias = -1.08 cm [95% CI -2.47 ; -0.30]; lower LoA = -6.36 cm [-8.78 ; -3.95]; upper LoA = 4.20 cm [1.78 ; 6.62]; SD of differences = 2.69 cm.

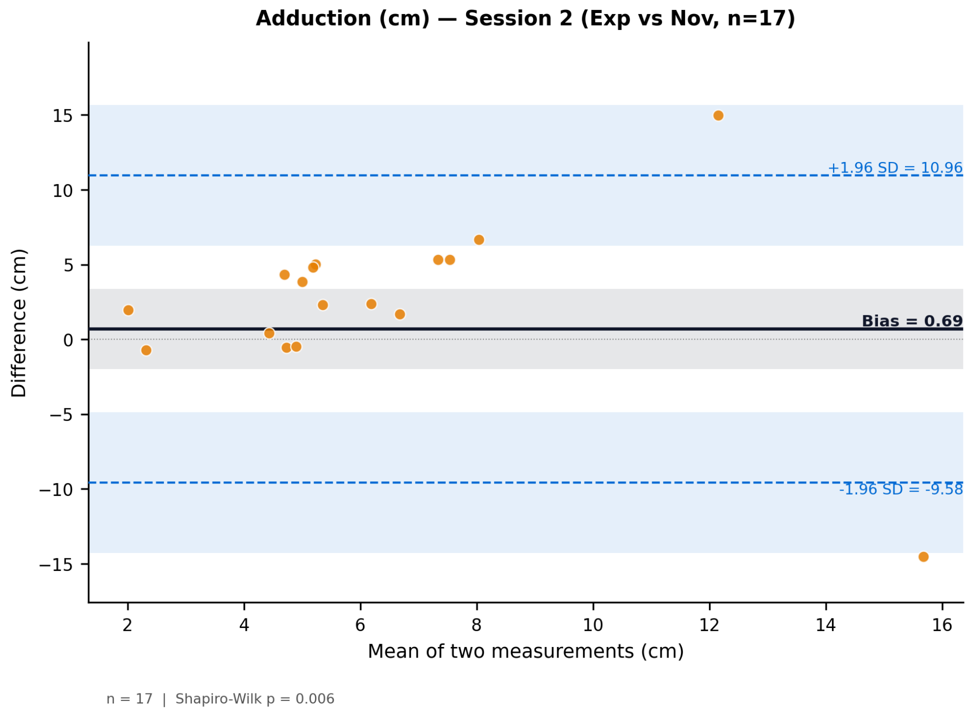

Figure S10. Bland–Altman plot of inter-rater agreement between the experienced and novice raters for adduction — Session 2 (n = 17). Mean bias = 0.69 cm [95% CI -2.00 ; 3.38]; lower LoA = -9.58 cm [-14.28 ; -4.88]; upper LoA = 10.96 cm [6.26 ; 15.65]; SD of differences = 5.24 cm.

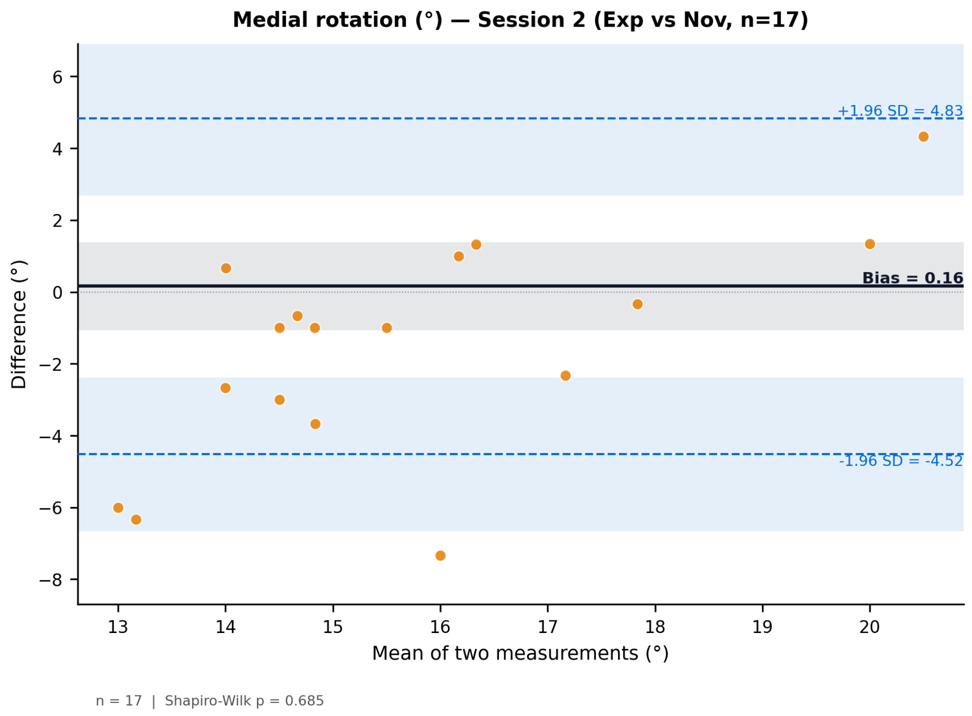

Figure S11. Bland–Altman plot of inter-rater agreement between the experienced and novice raters for medial rotation — Session 2 (n = 17). Mean bias = 0.16 ° [95% CI -1.07 ; 1.38]; lower LoA = -4.52 ° [-6.66 ; -2.38]; upper LoA = 4.83 ° [2.69 ; 6.97]; SD of differences = 2.39 °.

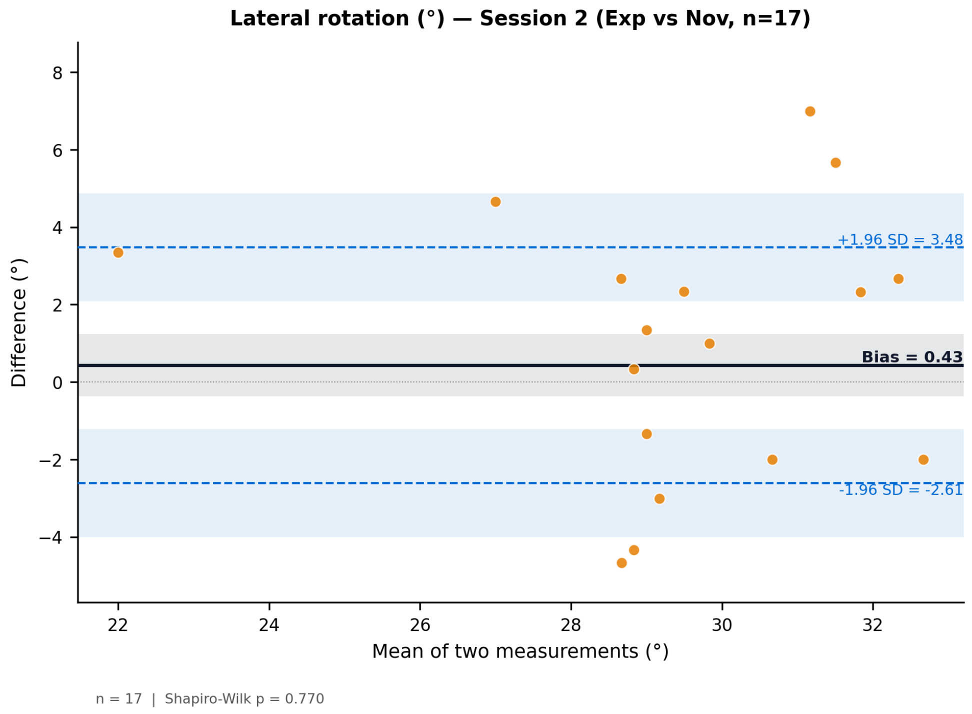

Figure S12. Bland–Altman plot of inter-rater agreement between the experienced and novice raters for lateral rotation — Session 2 (n = 17). Mean bias = 0.43 ° [95% CI -0.37 ; 1.23]; lower LoA = -2.61 ° [-4.01 ; -1.22]; upper LoA = 3.48 ° [2.08 ; 4.87]; SD of differences = 1.55 °.

Supplementary Material — Inter-rater agreement, Phase 2 (n = 57)

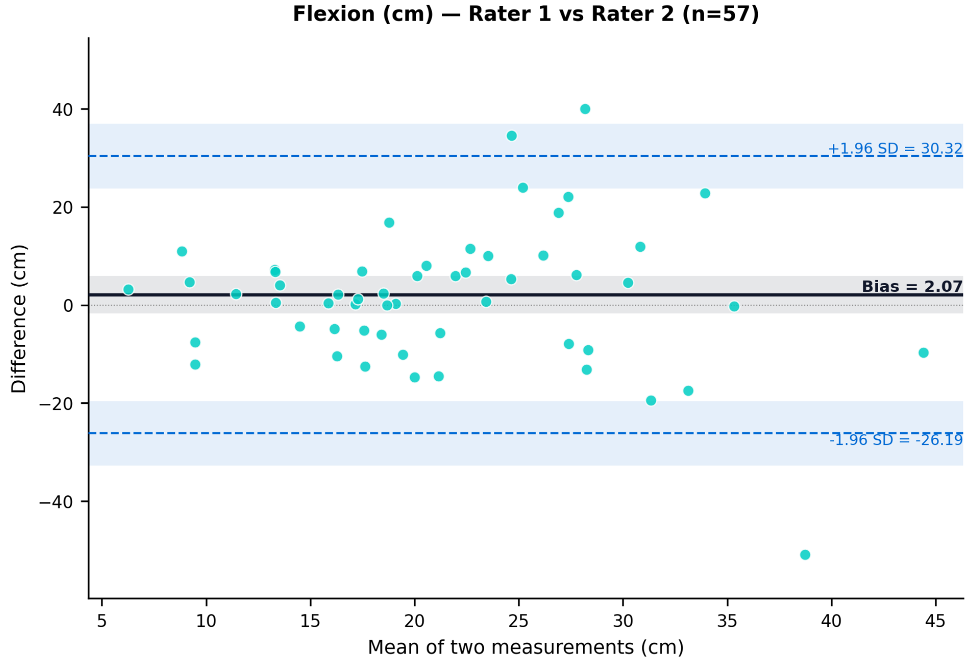

n = 57 | Shapiro-Wilk p = 0.009 (computed on n = 55 complete pairs)

Figure S13. Bland–Altman plot of inter-rater agreement between two novice raters for flexion (n = 57). Mean bias = 2.07 cm [95% CI -1.76 ; 5.89]; lower LoA = -26.19 cm [-32.77 ; -19.62]; upper LoA = 30.32 cm [23.75 ; 36.90]; SD of differences = 14.42 cm.

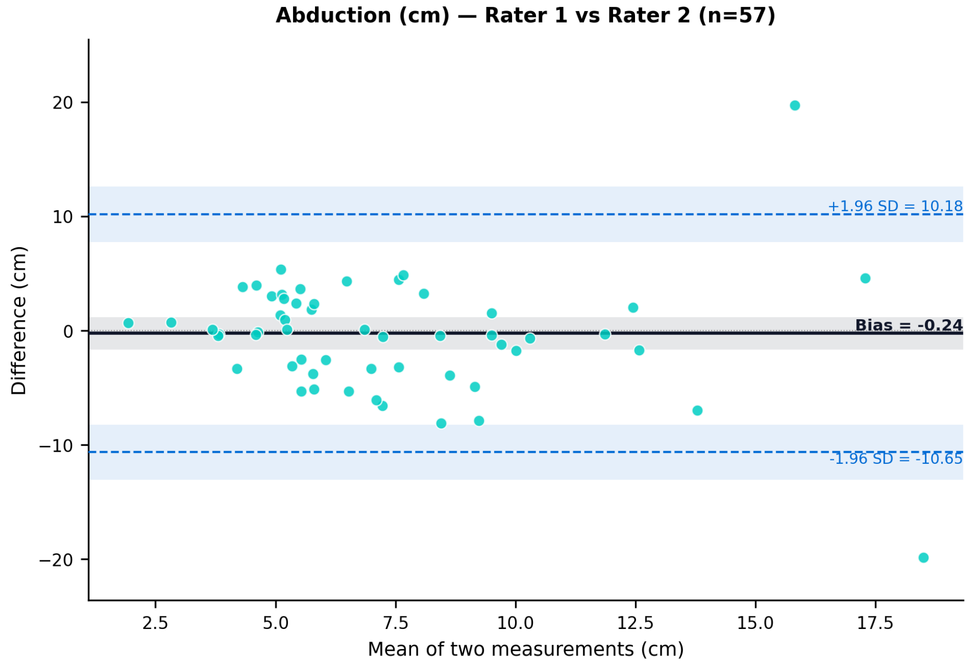

n = 57 | Shapiro-Wilk p = 0.000 (computed on n = 55 complete pairs)

Figure S14. Bland–Altman plot of inter-rater agreement between two novice raters for abduction (n = 57). Mean bias = -0.24 cm [95% CI -1.65 ; 1.17]; lower LoA = -10.65 cm [-13.08 ; -8.23]; upper LoA = 10.18 cm [7.76 ; 12.61]; SD of differences = 5.32 cm.

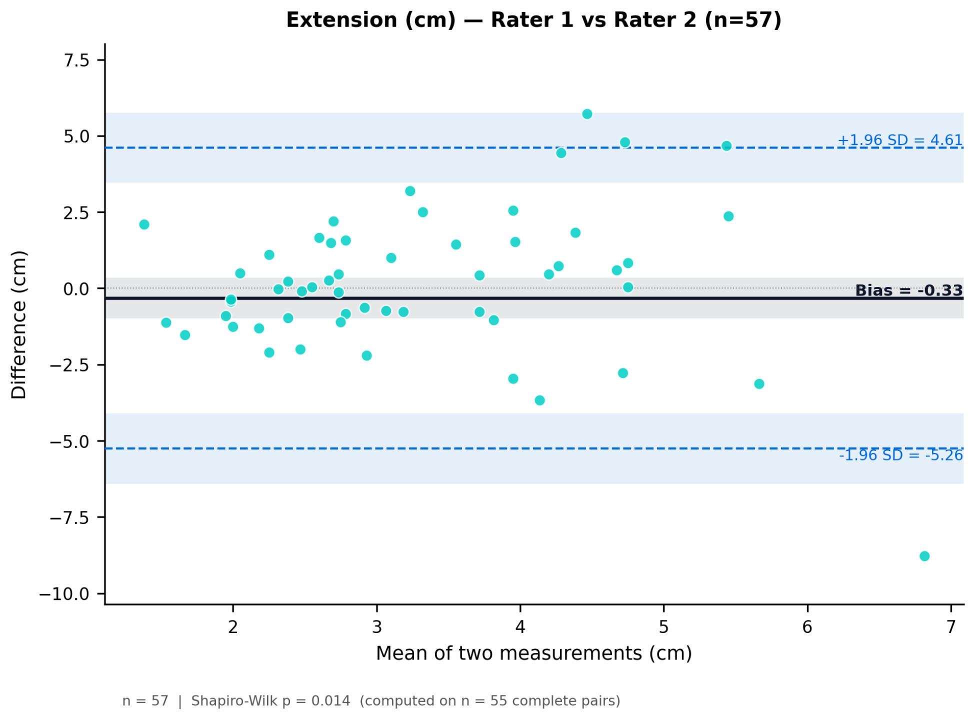

Figure S15. Bland–Altman plot of inter-rater agreement between two novice raters for extension (n = 57). Mean bias = -0.33 cm [95% CI -0.99 ; 0.34]; lower LoA = -5.26 cm [-6.41 ; -4.11]; upper LoA = 4.61 cm [3.46 ; 5.76]; SD of differences = 2.52 cm.

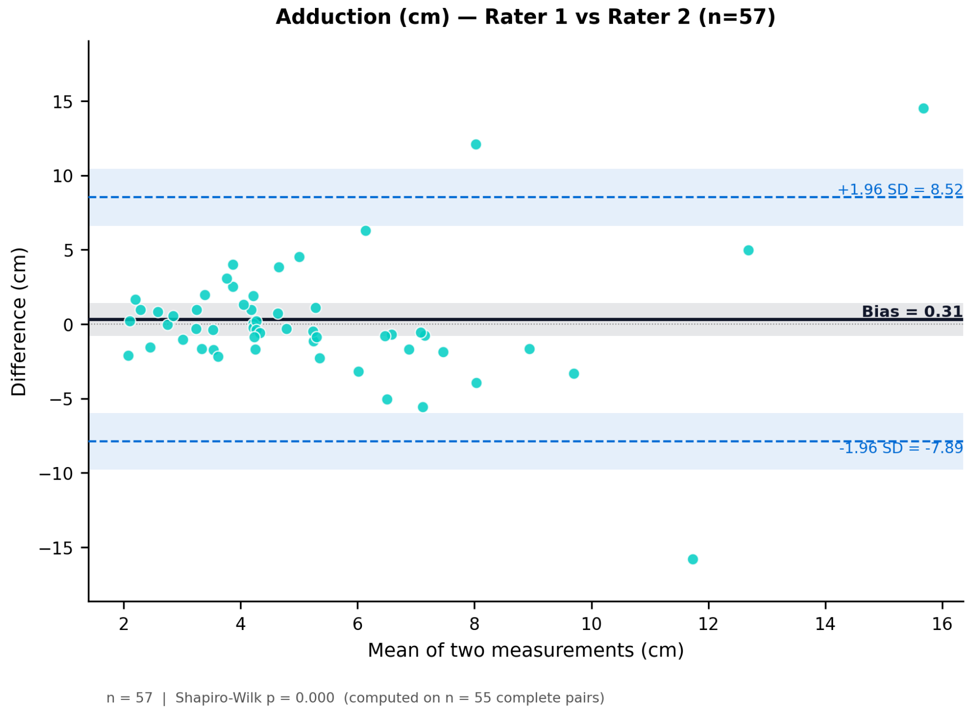

Figure S16. Bland–Altman plot of inter-rater agreement between two novice raters for adduction (n = 57). Mean bias = 0.31 cm [95% CI -0.80 ; 1.42]; lower LoA = -7.89 cm [-9.80 ; -5.98]; upper LoA = 8.52 cm [6.61 ; 10.43]; SD of differences = 4.19 cm.

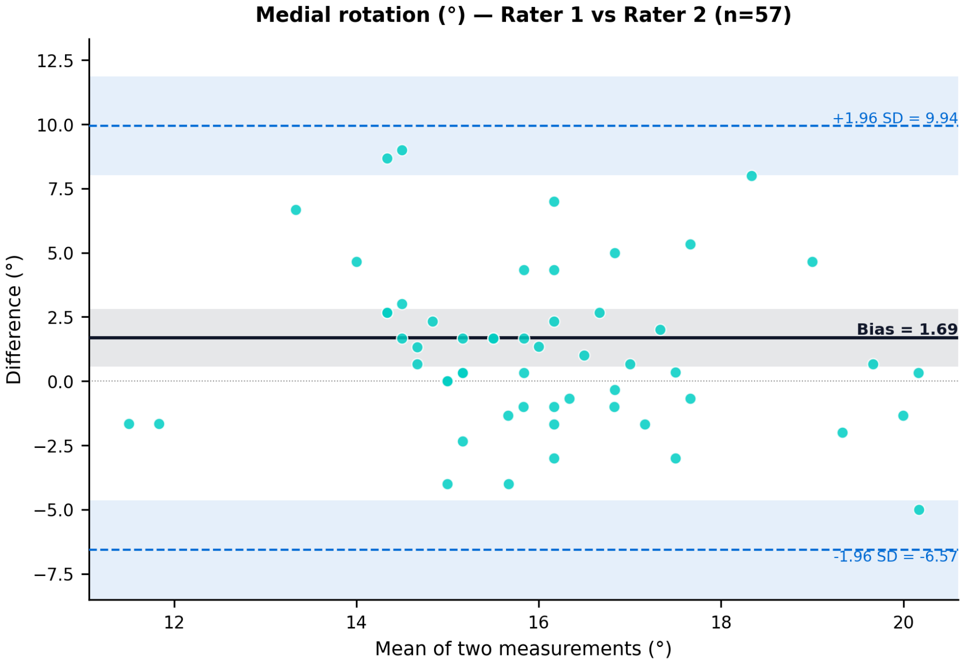

n = 57 | Shapiro-Wilk p = 0.106 (computed on n = 55 complete pairs)

Figure S17. Bland–Altman plot of inter-rater agreement between two novice raters for medial rotation (n = 57). Mean bias = 1.69 ° [95% CI 0.57 ; 2.81]; lower LoA = -6.57 ° [-8.48 ; -4.64]; upper LoA = 9.94 ° [8.02 ; 11.86]; SD of differences = 4.21 °.

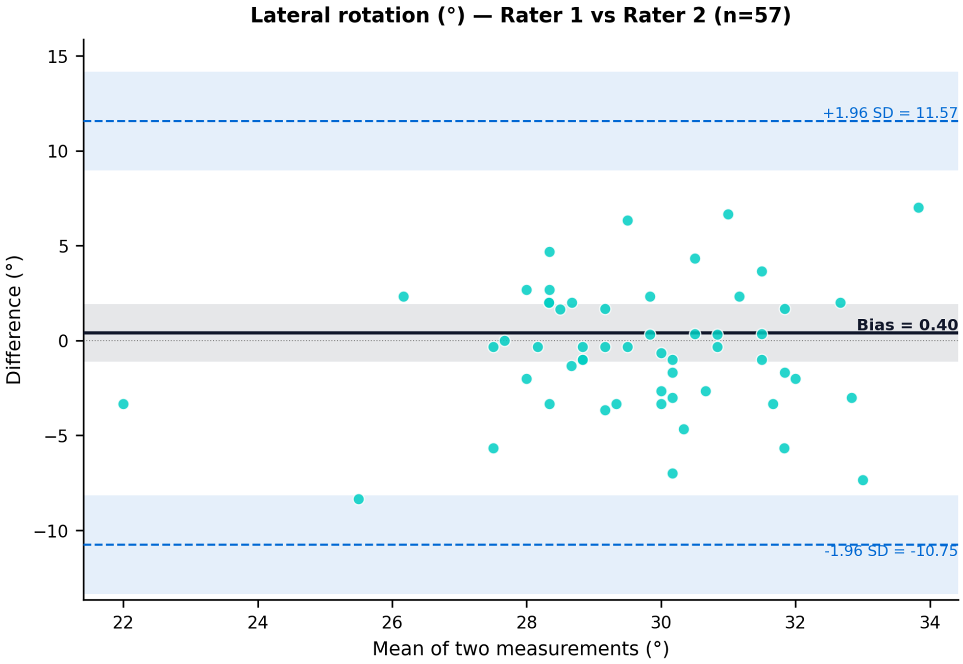

n = 57 | Shapiro-Wilk p = 0.667 (computed on n = 55 complete pairs)

Figure S18. Bland–Altman plot of inter-rater agreement between two novice raters for lateral rotation (n = 57). Mean bias = 0.40 ° [95% CI -1.11 ; 1.91]; lower LoA = -10.75 ° [-13.36 ; -8.15]; upper LoA = 11.57 ° [8.96 ; 14.16]; SD of differences = 5.69 °.
